# Protective Effects of Arginine on *Saccharomyces cerevisiae* Against Ethanol Stress

**DOI:** 10.1038/srep31311

**Published:** 2016-08-10

**Authors:** Yanfei Cheng, Zhaoli Du, Hui Zhu, Xuena Guo, Xiuping He

**Affiliations:** 1 CAS Key Laboratory of Microbial Physiological and Metabolic Engineering, Institute of Microbiology, Chinese Academy of Sciences, Beijing, China; 2 University of Chinese Academy of Sciences, Beijing, China

## Abstract

Yeast cells are challenged by various environmental stresses in the process of industrial fermentation. As the currently main organism for bio-ethanol production, *Saccharomyces cerevisiae* suffers from ethanol stress. Some amino acids have been reported to be related to yeast tolerance to stresses. Here the relationship between arginine and yeast response to ethanol stress was investigated. Marked inhibitions of ethanol on cell growth, expression of genes involved in arginine biosynthesis and intracellular accumulation of arginine were observed. Furthermore, extracellular addition of arginine can abate the ethanol damage largely. To further confirm the protective effects of arginine on yeast cells, yeast strains with different levels of arginine content were constructed by overexpression of *ARG4* involved in arginine biosynthesis or *CAR1* encoding arginase. Intracellular arginine was increased by 18.9% or 13.1% respectively by overexpression of *ARG4* or disruption of *CAR1*, which enhanced yeast tolerance to ethanol stress. Moreover, a 41.1% decrease of intracellular arginine was observed in *CAR1* overexpressing strain, which made yeast cells keenly sensitive to ethanol. Further investigations indicated that arginine protected yeast cells from ethanol damage by maintaining the integrity of cell wall and cytoplasma membrane, stabilizing the morphology and function of organellae due to low ROS generation.

*Saccharomyces cerevisiae* is an ideal model system for eukaryotic organisms, and moreover is an important biotechnologically applied yeast. In the process of industrial fermentation, *S. cerevisiae* might suffer various environmental stresses, such as fluctuation of temperature and pH, oxidative stress, osmotic stress and inhibitor stress, which have deleterious effects on both the cell growth and fermentation capability^1^. *S. cerevisiae* has attracted high interest for economical production of bioethanol due to its vast ability to synthesize ethanol from sugar. However, the increasing concentration of ethanol in fermentation broth becomes harmful to both cell growth and fermentation performance^2^,^3^. Change of membrane or cell wall compositions, induced expression of heat shock proteins, and accumulation of some stress protectants were observed in yeast cells in response to ethanol stress^4^.

Some amino acids have been reported to contribute to ethanol tolerance. Deletion of genes involved in tryptophan biosynthesis caused hypersensitivity of yeast cells to ethanol stress, while overexpression of some of those genes or addition of tryptophan to medium increased the ethanol tolerance^5^,^6^,^7^. Proline can stabilize proteins and membranes, and inhibit protein aggregation during refolding process, which makes it an effective protectant for yeast cells against various stresses^8^,^9^,^10^. Under ethanol stress, no obviously induced expression of genes involved in proline synthesis was observed, which suggests that yeast cells do not increase proline synthesis in response to ethanol stress^11^. However, deletion of *PRO1* involved in proline synthesis made yeast cells more sensitive to ethanol stress, while yeast cells with proline accumulation were found to be more tolerant to ethanol stress^7^,^12^. Arginine, which is associated closely with proline metabolism exhibits multiple functions *in vitro* for its particular chemical structure. It can inhibit heat-induced aggregation of partially folded protein intermediates and suppress protein-protein or protein-surface interactions during protein refolding and purification^13^,^14^,^15^,^16^. Actually, arginine has been used widely as an excipient in the protein-based biopharmaceuticals. Addition of arginine to medium provided significant protection for *Escherichia coli* against hydrogen peroxide-induced oxidative stress at pH 2.5, and it was found to be a compatible solute to improve the osmotic stress tolerance of *Candida glabrata*^17^,^18^. *S. cerevisiae* cells accumulated proline as well as arginine in the vacuole after freezing, and the survival rates of wild-type strain and the proline accumulating mutant after freezing were in proportion to intracellular arginine contents^19^. An arginase-defective yeast mutant accumulated a higher level of arginine and showed increased leavening ability during the frozen-dough baking process^20^. These results indicated that arginine might have a cryoprotective function in yeast. Under ethanol stress, intracellular arginine content in yeast cells remained constant during the detection period, which was six times of that in non-stressed yeast cells at 6 h^11^. However, the association between arginine content and the ethanol tolerance of yeast cells has not been investigated yet. Moreover, whether arginine has protective effects for yeast cells against other stresses remains unclear.

In this study, *S. cerevisiae* strains with various intracellular contents of arginine were constructed, and the correlation between intracellular arginine and stress tolerance was investigated. Moreover, the underlying possible mechanism for the role of arginine in ethanol tolerance was discussed.

## Results

### Growth and arginine metabolism of yeast cells under ethanol stress

**Figure 1:**
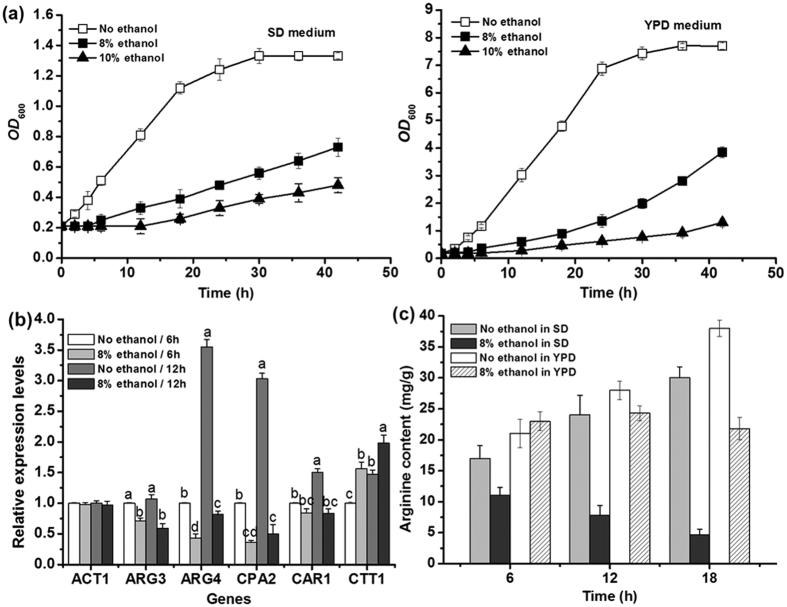
Effect of ethanol on cell growth and arginine metabolism. (**a**) Cell growth. (**b**) Transcriptional profiles of genes involved in arginine metabolism. Yeast cells were cultured in SD medium supplemented with histidine, leucine, tryptophan and uracil under stressed or non-stressed condition. *ACT1* was used as the control gene to normalize the expression level of different genes, while *CTT1* uppregulated by ethanol stress was also used a control gene. The relative expression level of each gene under non-stressed condition at 6 h was defined as a value of 1. Data are means ± SD (n = 3). Letters indicate significant differences among different treatments (p < 0.05, Duncan’s multiple-range test). (**c**) Arginine accumulation in the presence or absence of ethanol in SD medium or YPD medium. Data are means ± SD (n = 3).

Yeast cells of YS58 were cultured in YPD or SD medium supplemented with the required amino acids and uracil in the presence of different concentrations of ethanol (v/v). Under non-stressed conditions, strain YS58 displayed a relative long exponential growth period with a very short lag phase. Meanwhile, impaired growth under ethanol stress was observed in both YPD and SD media (Fig. 1a). To validate the inhibitory effect of ethanol on yeast cells, cultivations of yeast cells of YS58 in SD derived media, in which glucose concentration or concentrations of the supplemented compounds (His, Leu, Trp and Ura) changed, were conducted. Although the absolute values of biomass (*OD*_600_) at the same time point varied among different media, the cell growth or the inhibitory effect of ethanol on yeast cells displayed similar profiles (Figure S1). In the presence of 8% ethanol in SD medium, growth lag was 4 times of that in non-stressed condition, while growth rate and efficiency decreased 70.6% and 62.5% respectively when compared with those under non-stressed condition. The expression levels of genes involved in arginine metabolism and the intracellular content of arginine were monitored during cell growth in SD medium for 18 h. Under non-stressed condition, the expressions of *ARG4* encoding argininosuccinase, *CPA2* encoding carbamoyl-phosphate synthetase and *CAR1* encoding arginase were increased largely from 6 h to 12 h, which was consistent with the rapid cell growth during logarithmic phase. In the presence of 8% ethanol, only the expression of *ARG4* or *CPA2* was increased slightly from 6 h to 12 h, while expression of *CAR1* altered little during this period. For *ARG3* encoding ornithine carbamoyltransferase, no obvious difference in expression levels from 6 h to 12 h was observed either under stressed or non-stressed condition. However, inhibitory effects of ethanol on expression of genes involved in arginine biosynthesis or catabolism were observed, in which the expression levels of *CPA2* and *ARG4* were declined largely in the presence of ethanol (Fig. 1b). The intracellular arginine content decreased largely under ethanol stress probably due to the significant reduction of expression of genes *CPA2* and *ARG4* (Fig. 1c). In YPD medium, similar expression profiles as in SD medium were observed for arginine metabolic genes. However, the intracellular arginine accumulation under ethanol stress did not change largely during the detection period, but which was much lower at 12 h and 18 h than that under non-stressed condition (Fig. 1c). These results indicate that ethanol appears to perturb arginine metabolism in *S. cerevisiae*.

### Extracellular addition of arginine promotes cell growth under ethanol stress

**Figure 2:**
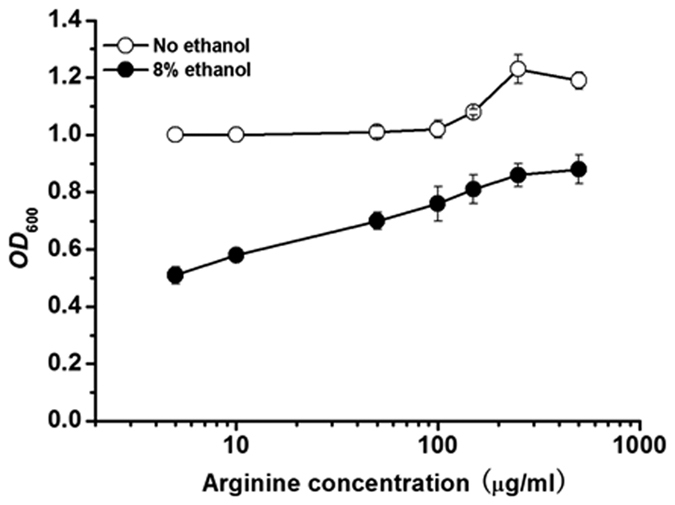
Effect of extracellular addition of arginine on cell growth under ethanol stressed or non-stressed condition in SD medium. Yeast cells were cultured in SD media supplemented with histidine, leucine, tryptophan, uracil and different concentrations of arginine under ethanol stressed or non-stressed conditions for 24 h. *OD*_600_ of strain YS58 under non-stressed condition without supplement of arginine was definied as 1. Data are means ± SD (n = 3).

To determine whether the decrease of arginine content in yeast cells plays roles in inhibitory effect of ethanol on cell growth, the influence of extracellular addition of arginine on cell growth (*OD*_600_) was monitored under stressed or non-stressed condition (Fig. 2). In the absence of ethanol, arginine had almost no effect on cell growth of YS58 at concentrations less than 150 μg ml^−1^ (0.86 mM), and then only 23.1% increase of cell growth was obtained when arginine was added at a concentration of 250 μg ml^−1^ (1.44 mM). Under ethanol stress, however, obviously positive effect was observed for arginine on cell growth. After cultivation in the presence of 8% ethanol for 24 h, cell density was decreased by 62.9% when compared with that under non-stressed condition. When arginine was added in medium at concentration of 250 μg ml^−1^ under ethanol stress, a cell density equaled to 86.3% of that without addition of arginine under non-stressed condition was observed, which was 2.3-fold of that under ethanol stressed condition without addition of arginine. These results suggest that arginine has potential roles to protect yeast cells from ethanol stress.

### Intracellular accumulation of arginine protects yeast cells against ethanol stress

**Figure 3:**
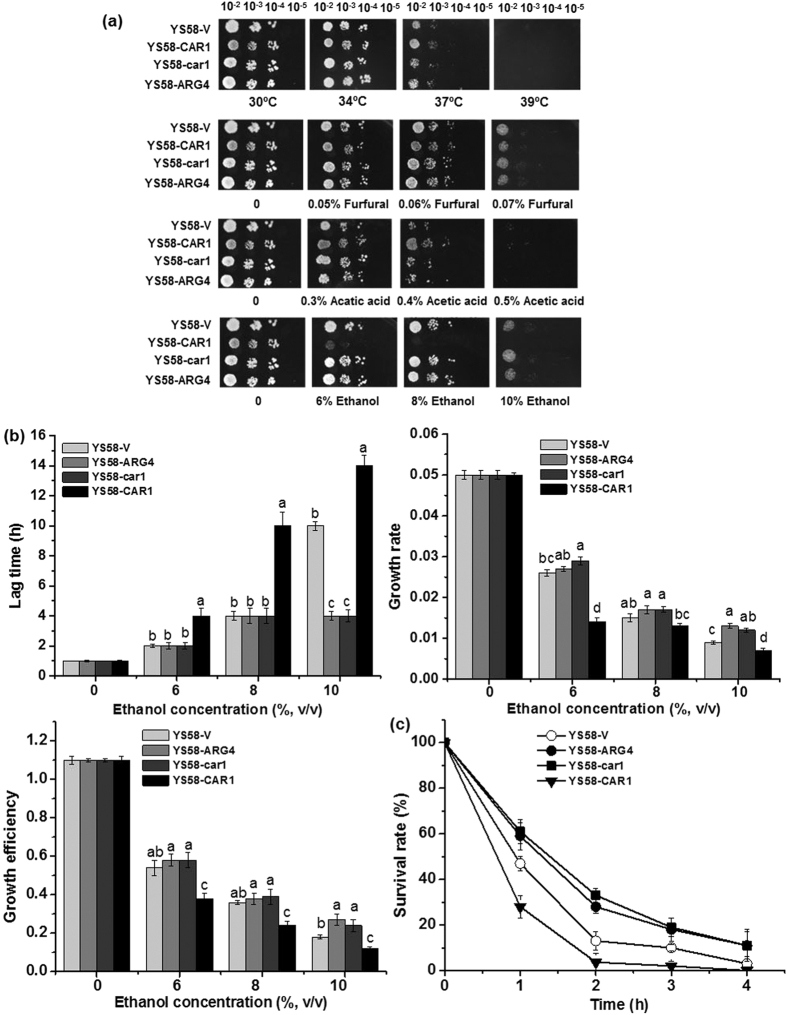
Response of yeast cells with different accumulation of arginine to ethanol stress. (**a**) Growth of yeast cells on YPD solid media under various stressed conditions by spot dilution assay. (**b**) Comparison of cell growth in SD medium containing the required amino acid and uracil with or without ethanol by liquid growth assay. Lag time, growth rate and growth efficiency were used to characterize cell growth. (**c**) Survival assay of different yeast strains under 15% ethanol stress. Data are means ± SD (n = 3). Letters indicate significant differences among different treatments (p < 0.05, Duncan’s multiple-range test).

To further clarify the correlation between arginine metabolism and response to ethanol stress, yeast strains with different levels of arginine accumulation were constructed by overexpression or disruption of genes involved in arginine biosynthesis (*ARG4*) or catabolism (*CAR1*). Intracellular content of arginine was increased by 18.9% or 13.1% through overexpression of *ARG4* or disruption of *CAR1*, while overexpression of *CAR1* caused a significant decrease (41.1%) in arginine content (Table 1). Meanwhile, 25.6% increase in ornithine content and 26.5% increase in proline content occurred in *CAR1* overexpressing yeast strain. Cell growth of yeast strains was compared on solid YPD medium under various stress conditions. No significant difference in cell growth was observed among these yeast strains under heat, furfural or acetic acid stress, while the growth of YS58-CAR1 with low arginine accumulation was strongly inhibited under ethanol stress (Fig. 3a). Same results were obtained when SD medium was used.

To validate the results of spot assay, liquid cultivations in the presence of different concentrations of ethanol were performed. There was no obvious growth difference among yeast strains YS58-V, YS58-car1, YS58-ARG4 and YS58-CAR1 in the absence of ethanol. However, significant differences in lag time, growth rate and growth efficiency were observed among different yeast strains in the presence of ethanol. Yeast strain YS58-CAR1 with the least arginine accumulation displayed the longest lag time, the lowest growth rate and growth efficiency under ethanol stressed conditions, while yeast strains YS58-ARG4 and YS58-car1 with higher intracellular accumulation of arginine gave much shorter lag time and higher growth rate and growth efficiency than strains YS58-V and YS58-CAR1 (Fig. 3b). In further survival assay, higher survival rate was obtained for strains with higher arginine accumulation in the presence of 15% ethanol. After 2 h under ethanol stress, the survival rates of YS58-ARG4 and YS58-car1 were 2.15 and 2.54 times of that of control strain YS58-V, while the survival rate of strain YS58-CAR1 was decreased by 71.5% in comparison with the control strain (Fig. 3c). These results confirm that proper levels of arginine accumulation play an important role for yeast cells in ethanol tolerance.

### Protective effect of arginine on cytoplasma membrane integrity

**Figure 4:**
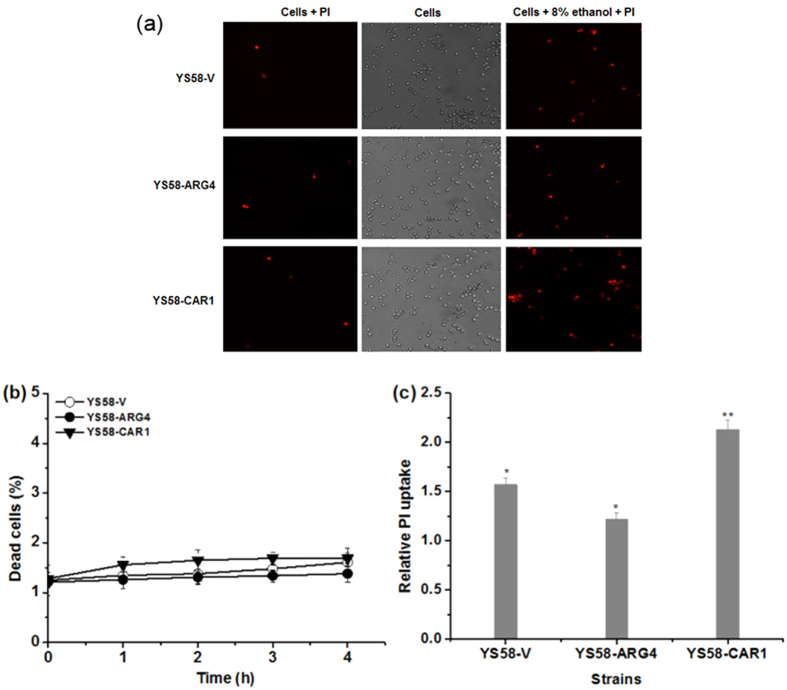
Membrane integrity analysis of different yeast strains. (**a**) Representative images of PI uptake observed by fluorescence microscopy. (**b**) Determination of dead cell rates under 8% ethanol stress by flow cytometric analysis. (**c**) Determination of PI uptake under 8% ethanol for 1 h by fluorospectrophotometer. The level of PI uptake was expressed as the ratio of relative fluorescence units (RFU) under ethanol stressed and non-stressed conditions. Yeast cells were cultured in SD medium supplemented with histidine, leucine, tryptophan and uracil. Data are means ± SD (n = 3). Asterisks indicate significant differences between different strains (*P < 0.05; **P < 0.01; Student’s t-test).

To seek the protective mechanism of arginine on yeast cells under ethanol stress, the cytoplasma membrane integrity in the presence or absence of 8% ethanol was determined by PI uptake analysis. Under non-stressed condition, only 1–2% of the yeast cells were stained by PI, and no obvious difference was observed among different strains. However, after 1 h in the presence of 8% ethanol, yeast cells stained by PI increased largely (Fig. 4a). Very low mortality of cells (1–2%) was observed by flow cytometer in the presence of 8% ethanol for yeast strains with various arginine accumulations (Fig. 4b), which will eliminate the interference of dead cells on membrane integrity analysis. In the further fluorescence intensity analysis, a 22.3% decrease in the relative PI uptake was observed in strain YS58-ARG4 with higher arginine content, while YS58-CAR1 with lower arginine accumulation showed a 35.7% increase in relatve PI uptake compared with that of control strain YS58-V under ethanol stress (Fig. 4c). These results suggest that arginine can protect yeast cells from ethanol stress by reducing cell membrane damage.

### Protective effects of arginine determined by transmission electron microscopy (TEM) analysis

**Figure 5:**
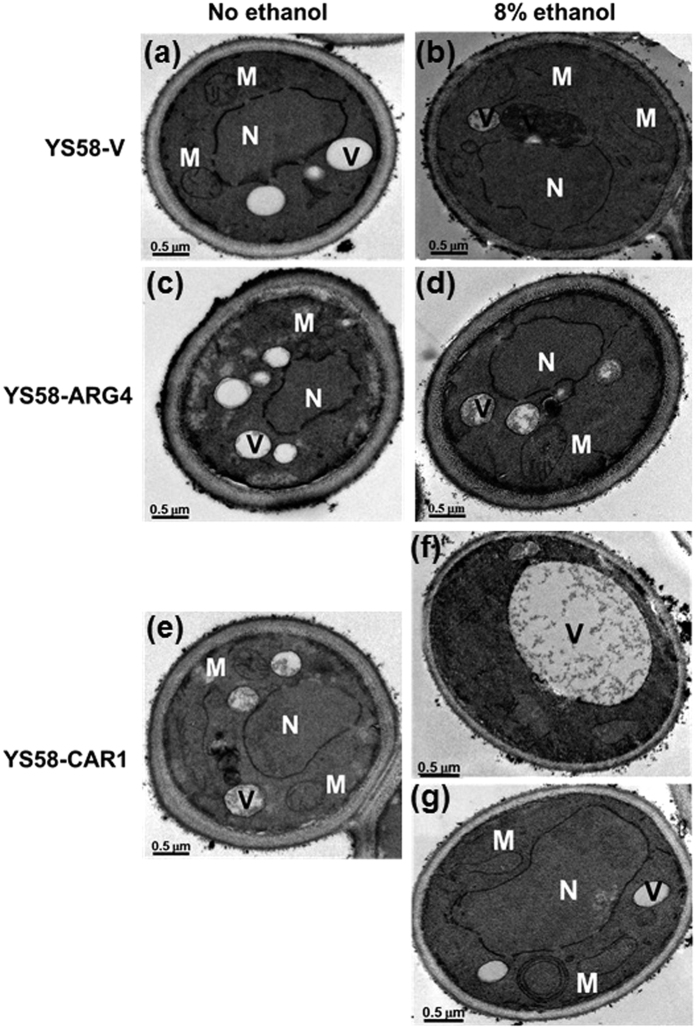
Transmission electron microscopy (TEM) analysis for determination of cellular damages caused by ethanol. Yeast cells grown in SD medium supplemented with histidine, leucine, tryptophan and uracil with either no ethanol or 8% ethanol for 8 h were fixed and thin-sectioned for TEM analysis. (**a**) YS58-V without ethanol. (**b**) YS58-V with 8% ethanol. (**c**) YS58-ARG4 without ethanol. (**d**) YS58-ARG4 with 8% ethanol. (**e**) YS58-CAR1 without ethanol. (**f** and **g**) YS58-CAR1 with 8% ethanol. M: mitochondria, N: nucleus, V: vacuole.

The cellular damages induced by ethanol were tested by TEM analysis for yeast strains with different contents of arginine. Under non-stressed condition, typical morphologies were observed for mitochondria, vacuole and nucleus in yeast cells. Mitochondria were evenly distributed in the cell periphery, and vacuoles were relatively small and disperse (Fig. 5a, 5c and 5e). Moreover, no obvious morphological differences in organellae were observed among different yeast strains. When yeast cells were treated with 8% ethanol for 8 h, some morphologic changes were observed. Mitochondria appeared swollen and lengthened with less structured cristae in cells of control strain YS58-V under ethanol stress (Fig. 5b), while no obvious metamorphotic mitochondria were observed in cells of strain YS58-ARG4 with higher arginine accumulation (Fig. 5d). Vacuole and nucleus in yeast cells of YS58-V and YS58-ARG4 seemed little affected by 8% ethanol treatment. For strain YS58-CAR1 with less arginine accumulation, however, both mitochondria and nucleus appeared highly swollen, and segregated vacuolar structures aggregated into a single and large vacuole in a large number of cells under ethanol stress (Fig. 5f and 5g). Moreover, the external cell wall appeared thinned in yeast cells of YS58-V and YS58-CAR1 treated by ethanol when compared with yeast cells under non-stressed condition. These results suggested that damage in cell wall and membrane occurred in the presence of ethanol, and then changes in intracellular environment caused by ethanol uptake impaired the structure and function of organellae. For yeast cells with higher arginine accumulation, however, lesser damages in cell wall, membrane and organellae occurred under ethanol stress.

### ROS generation in different yeast strains

**Figure 6:**
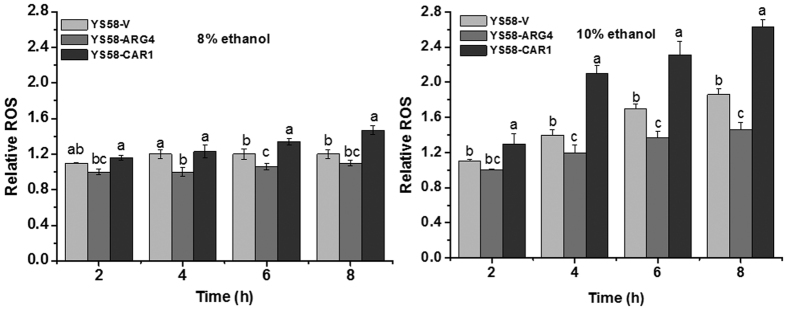
ROS generation of different yeast strains under ethanol stress. SD medium supplemented with histidine, leucine, tryptophan and uracil was used for cell culture. Relative ROS levels were used to indicate the changes of ROS generation in different yeast strains under ethanol stress with respective to the untreated yeast cells. Data are means ± SD (n = 3). Letters indicate significant differences among different treatments (p < 0.05, Duncan’s multiple-range test).

Yeast cells were cultured in the absence or presence of 8% or 10% ethanol for different hours. ROS generation in different yeast cells was monitored by flow cytometry using DHR123 as the ROS indicator. Relative ROS levels were used to indicate the changes of ROS generation in different yeast strains under ethanol stress with respective to the untreated yeast cells. ROS generation occurred in ethanol treated cells, and which was more apparent in the presence of 10% ethanol (Fig. 6). Moreover, yeast strain YS58-ARG4 with higher arginine accumulation produced less ROS than the control strain YS58-V, while the less arginine-accumulating strain YS58-CAR1 generated much higher ROS under ethanol stress. With treatment by 10% ethanol for 8 h, relative ROS level of YS58-CAR1 was 1.4 and 1.8 times of that of YS58-V and YS58-ARG4. These results indicated that ROS accumulation in *S. cerevisiae* is caused by ethanol stress, however, this effect is weakened by arginine, which confirms that reduction of ROS generation in arginine-accumulating yeast cells is one of the mechanisms of arginine to protect yeast cells from ethanol stress.

## Discussion

The existence of higher-titer of ethanol in fermentation broth is one of the challenges that yeast cells have to face during the bioethanol production process. Response of yeast cells to ethanol stress and the mechanisms of ethanol tolerance involve in complex interplays of various pathways and network events^4^. It has been reported that ethanol appears to perturb the metabolic profile of amino acids in *S. cerevisiae*. The increase of intracellular amount of Pro, Ser, Gly, Ala, Val, His and Lys, as well as decrease of intracellular Glu content and maintenance of Arg concentration, occurred in yeast cells treated by ethanol^11^. Here, inhibitory effects of ethanol on cell growth, expression of genes involved in arginine metabolism and intracellular accumulation of arginine were observed. In YPD medium, the intracellular arginine accumulation changed little during exposure to ethanol, which might be caused by the uptake of arginine from YPD medium due to the increase of membrane permeability or expression of genes encoding transporters under ethanol stress^11^. In the SD medium, the interference of extracellular amino acids was eliminated, so the influence of ethanol on metabolic profiles of amino acids could be interpreted by the change of intracellular content.

The inhibition of ethanol on both arginine biosynthesis and cell growth suggested that arginine, like proline or tryptophan^4^, might be related to ethanol tolerance. To address their relationship, effect of extracellular addition of arginine on yeast cell growth was tested under ethanol stressed or non-stressed conditions. Positive influence of arginine on cell growth was observed under both conditions, and much stronger protective function occurred in the presence of ethanol. For further confirmation of the protective effect of arginine, yeast strains with higher or lower intracellular arginine accumulation were constructed by overexpression or deletion of genes involved in arginine biosynthesis and degradation. Overexpression of *ARG4* or deletion of *CAR1* increased the accumulation of arginine in yeast cells, which made yeast cells more tolerant to ethanol than the control cells, while decrease of intracellular arginine accumulation in the *CAR1* overexpressing strain caused yeast cells more sensitive to ethanol stress (Table 1, Fig. 3). It was reported that enhancement of intracellular proline accumulation improved ethanol tolerance of sake yeast strain^12^. In *CAR1* overexpressing yeast strain, however, although the intracellular proline content was increased by 26.5% compared to the control strain, the former strain exhibited much higher ethanol sensitivity than the control strain. This result is consistent with the report of Takagi *et al*.^21^. The significant decrease (41.1%) of intracellular arginine content in *CAR1* overexpressing yeast strain might be the main reason of ethanol sensitivity compared to control strain YS58-V. These results indicate that arginine or synergistic action of arginine and proline plays more important roles than proline alone in ethanol tolerance.

Gene deletion assays suggest that many genes related to cell wall composition or organization are associated with yeast tolerance to ethanol stress^2^,^5^,^7^,^22^, and remodeling of yeast cell wall occurs under ethanol stress^22^. Relative thinner cell wall was observed for the control strain YS58-V and low arginine-accumulating strain YS58-CAR1 in the presence of ethanol, while the thickness of cell wall of YS58-ARG4 with higher arginine content appeared slight difference between ethanol stressed and non-stressed conditions (Fig. 5). It seems that arginine plays some roles in maintenance of cell wall integrity. Changes of membrane composition and remodeling of membrane structure occurred under ethanol stress, which might influence the fluidity and permeability of plasma membrane^4^,^23^. In this study, although no obvious differences in plasma membrane were observed by TEM analysis for different yeast strains in the absence or presence of ethanol, increase in membrane permeability occurred under ethanol stress, which indicated that membrane integrity was disrupted (Fig. 4). However, the deficits of membrane caused by ethanol were inversely proportional to the intracellular arginine content, which suggests that arginine possesses protective function for plasma membrane from ethanol damage.

In addition to deficits of membrane and cell wall, internal cellular damages, such as swelling and elongating of mitochondria, aggregation of segregated vacuolar structures into a single and large vacuole and irregular nucleus, were also observed for yeast cells exposing to ethanol^24^,^25^,^26^,^27^. Changes in morphologies of vacuoles, mitochondria or nucleus under ethanol stress were also observed in this study. However, much severer damages caused by ethanol occurred in yeast cells with low arginine content than in those of control strain YS58-V. Moreover, no obvious influence of ethanol on morphologies of cytoplasmic organelles was detected for yeast cells with higher accumulation of arginine. As the degradative organelle and major storage compartment, vacuole is a highly responsive structure whose morphology and functions are highly dynamic in response to different extracellular or intracellular stimuli. In actively growing cells, vacuoles exist mainly in medium-sized fragmented form, while yeast cells in stationary phase or under some stressed conditions are characterized by a grossly enlarged vacuole^24^,^26^. Vacuole morphology is dictated by the equilibrium between fusion and fission of vacuolar membranes^25^. Although the exact regulatory mechanism and physiological function of vacuolar morphological alteration are not yet clear, the big single vacuole phenotype might imply defect for vacuole fission that is necessary for cell division and active growth. So, occurrence of an enlarged swelling vacuole indicates the decrease of cell viability. In yeast cells with higher arginine accumulation, no obvious difference in vacuole morphology was observed between ethanol stressed and non-stressed conditions, which suggests that arginine can reduce the cellular damages caused by ethanol by maintaining the intracellular physiological environment relative stable.

It has been reported that the accumulation of reactive oxygen species (ROS) can be increased under ethanol stress, which are known to cause cellular damages^28^,^29^,^30^. To evaluate whether the protective effects of arginine on yeast cells against ethanol stress are associated with ROS generation, the mitochondrial ROS levels were compared among yeast strains with different arginine accumulation under ethanol stressed and non-stressed conditions. As indicated in Fig. 6, much higher ROS accumulation was observed in strain YS58-CAR1 than in the control strain YS58-V and the arginine accumulating strain YS58-ARG4 under ethanol stress when compared with non-stressed condition, which is very apparent in the presence of 10% ethanol. These results indicated that arginine could reduce cellular oxidative stress and protect yeast cells from damage caused by ethanol stress. It has been reported that the gradual accumulation of ethanol during the alcohol fermentation process provoked the ROS generation, which might be responsible for the inhibition on cell viability and the reduction of final ethanol titer^30^. Hence, yeast strains with higher intracellular arginine accumulation might be superb for ethanol production due to low ROS generation under ethanol stress, especially in the high gravity fermentation process. The ethanol productivity of arginine-accumulating yeast strain should be investigated in detail in future studies.

In conclusion, ethanol damages on external cell wall, plasma membrane and intracellular organellae were detected in the present study, which is consistent with the previous reports. However, in arginine accumulating yeast strain, such damages were attenuated largely due to low ROS generation, which results in ethanol tolerance. The specific molecular structure and the ability to form multiple hydrogen bonds make arginine an effective protectant for proteins, DNA and phospholipids to maintain intracellular homeostasis^15^,^16^,^31^, which might be the major underlying mechanism for arginine to protect yeast cells against cellular damages from ethanol stress. So, arginine, just like trehalose, can be a stress protectant for *S. cerevisiae* against ethanol, and modulation of the intracellular arginine levels should be considered in the future development of ethanol tolerant strains. Moreover, arginine can be converted to nitric oxide (NO) by nitric oxide synthase (NOS), which has been regarded as one of the underlying mechanisms of protective effect of arginine on mammalian cells^31^. Although mammalian or bacterial NOS orthologues is not found in the genome of yeast, NOS-like activity was detected in *S. cerevisiae*^32^,^33^, and it was found that NO production from arginine or other NO donors conferred heat-stress tolerance on yeast cells^34^. In this study, the accumulation of arginine in yeast cells overexpressing *ARG4* or disruption of *CAR1* might enhance the NO production, which contributes to ethanol tolerance. In *CAR1* overexpressing yeast cells, competitive consumption of arginine by arginase might limit the synthesis of NO to make yeast cells sensitive to ethanol. However, it has also been suggested that NO may enhance intracellular ROS generation^33^,^35^,^36^, which is inconsistent with the results obtained in this study. As a signal molecule, the NO production might be an important part of the mechanisms of yeast response to stresses, but its physiological roles under different stressed conditions might vary and require to be investigated in detail.

## Methods

### Strains, plasmids, primers and growth conditions

Strains and plasmids used in this study are listed in the supplementary Table S1. The oligonucleotide primers used in this study are listed in the supplementary Table S2. *S. cerevisiae* YS58 was used as the host for overexpression or disruption of genes involved in arginine metabolism^37^. *S. cerevisiae* CE25 (China General Microbiological Culture Collection Centre, CGMCC 2.1418) was used as the source of some selective marker genes. *Escherichia coli* DH5α was used as a general host for plasmid propagation. Yeast cells were grown generally at 30 °C in YPD medium^38^. To screen or analyze yeast transformants, minimal synthetic defined (SD) medium (0.67% yeast nitrogen base without amino acids, 1% glucose) supplemented with or without 30 mg l^−1^ each of uracil, leucine, histidine or 90 mg l^−1^ of tryptophan was also used. For analysis of inhibitor tolerance, acetic acid, ethanol or furfural was added into the YPD or SD medium after the medium was sterilized by autoclavation and cooled to about 50 °C. *E. coli* DH5α was grown at 37 °C in Luria-Bertani (LB) medium^39^. When necessary, 100 μg ml^−1^ of ampicillin was added to LB medium.

### General DNA manipulations

General DNA manipulations in *E. coli* or *S. cerevisiae* were performed according to standard methods^38^,^39^. Polymerase chain reaction (PCR) was conducted using high fidelity DNA polymerase KOD plus according to the manufacturer′s instruction (TOYOBO, Japan). Purification of DNA fragments was performed using PCR Clean-up kit or DNA Gel Extraction kit (Axygen scientific Inc, USA). DNA sequencing was completed by Shanghai Invitrogen Biological Technology CO., LTD. Total RNA was isolated by using the hot phenol method^38^. Gene expression was analyzed by qRT-PCR using the Quant one-step qRT-PCR kit (SYBR Green) with *ACT1* as a control.

### Construction of recombinant yeast strains

A 1350 bp DNA fragment covering the coding region of arginase gene *CAR1* (Accession number M10110) and its upstream and downstream regions was amplified from the genomic DNA of *S. cerevisiae* YS58 by PCR with primer pair CAR1-F1/CAR1-R1. The amplified fragment was digested with *Kpn*I/*Sal*I, and then inserted into the corresponding sites of plasmid pUC18 to generate pUC18C1. Phosphoribosylanthranilate isomerase gene *TRP1* (Accession number NC_001136) was amplified from the genomic DNA of *S. cerevisiae* CE25 with primer pair TRP1-F/TRP1-R. After digested with *Afl*II/*Eco*NI, *TRP1* was inserted into the corresponding sites of plasmid pUC18C1 to generate recombinant plasmid pUc1T1, in which 692 bp coding sequence of *CAR1* was replaced by 1067 bp of *TRP1*. Disruption cassette *car1*::*TRP1* was amplified from plasmid pUc1T1 with primer pair CAR1-F1/CAR1-R1, and transformed into *S. cerevisiae* YS58 to disrupt the chromosomal *CAR1* by double-crossover homologous recombination. Transformants were screened using *TRP1* as the selectable marker. The disruption of *CAR1* was confirmed by PCR and sequence analysis. The resulting mutant was designated as yeast strain YS58-car1.

The geneticin (G418) resistance cassette was amplified from plasmid pFA6a-kanMX4^40^ with primer pair KanMX-F/KanMX-R, and inserted into plasmid YCp50^41^ after digested by *Sal*I/*Ap*aI to generate plasmid pYCPG. The *ADH1* promoter was amplified from *S. cerevisiae* YS58 genomic DNA with primer pair ADH1-F/ADH1-R, and then inserted into the *Eco*RI and *Bam*HI sites of pYCPG to generate plasmid pYCPGA1. For overexpression of *CAR1* or *ARG4*, the DNA fragment covering the coding region and its terminator region of *CAR1* or *ARG4* (Accession number K01813) was amplified from *S. cerevisiae* YS58 genomic DNA with primer pair CAR1-F2/CAR1-R2 or ARG4-F1/ARG4-R1. The amplified DNA fragments were digested by *Bam*HI/*Sa*lI, and then inserted into the corresponding sites of pYCPGA1 to generate plasmid pYGAC1 or pYGAA4 respectively, in which the expression of *CAR1* or *ARG4* was driven by *ADH1* promoter. Plasmid pYGAC1 or pYGAA4 was introduced respectively into *S. cerevisiae* YS58 to generate recombinant strain YS58-ARG4 or YS58-CAR1. As a control, *S. cerevisiae* YS58 was transformed with vector pYCPGA1 to generate control strain YS58-V. All PCR products were verified by DNA sequencing. All yeast strains were confirmed by G418 resistance and PCR analysis. The genetic stability of yeast strains was analyzed as described previously^42^.

### Stress tolerance assay

Tolerance of yeast strains to different stresses were compared by spot dilution assay on solid media first. After cultured in YPD medium at 30 °C for 18 h, yeast cells were harvested, washed twice with distilled water and resuspended in distilled water at a same cell concentration (*OD*_600_ = 0.1). The cell suspension was diluted serially and kept at room temperature for 2 h. 5 μl of each 10-fold dilution (10^−2^–10^−5^) was spotted onto YPD plates supplemented with different concentrations of acetic acid (0.3%, 0.4% and 0.5%, v/v), ethanol (6%, 8% and 10%, v/v) or furfural (0.05%, 0.06% and 0.07%, v/v). The growth of yeast cells was assessed after incubation at 30 °C or other temperatures for 48 h. For analysis of acetic acid tolerance, the pH of YPD medium, about 5.5 in general, was adjusted to 4.5 with 2 M HCl. Assays of stress tolerance were also performed on SD plates supplemented with uracil, leucine, histidine and tryptophan.

Ethanol tolerance was also analyzed by liquid growth assay. Yeast cells precultured in YPD at 30 °C with an agitation of 200 rpm for 18 h were inoculated into 50 ml of YPD or SD media supplemented with uracil, leucine, histidine and tryptophan to a final cell density equivalent to 0.2 of *OD*_600_. Ethanol was added to the cell suspension at final concentration of 8% or 10% (v/v) respectively and then cultivated at 30 °C with an agitation of 200 rpm. The cell growth was monitored periodically. Three fundamental growth variables, growth lag (the intercept of the initial density and the slope), growth rate (the slope of the exponential phase of the growth curve, between 12 and 24 h) and growth efficiency (the total change in density for cells reached 30 h)^43^, were used to characterize the ethanol sensitivity of yeast strains and the protective effect of arginine.

Survival assay was also performed to characterize the ethanol tolerance of different yeast strains. From a preculture, a 1% (v/v) inoculum was added to 10 ml of fresh SD medium supplemented with the required amino acids and uracil and then incubated at 30 °C for 12 h. Yeast cells were harvested by centrifugation at 3000× *g* for 5 min, washed twice with sterile 100 mM phosphate buffer saline (PBS, pH 7.4) and resuspended in PBS to a final cell concentration equivalent to 0.8 of *OD*_600_. 177 μl of ethanol was added to 1 ml of the cell suspension to a final concentration of 15% (v/v), and followed by incubation at 30 °C with an agitation of 200 rpm. Yeast cells were harvested from samples drawn at different time and resuspended in 1 ml of PBS. Survival was measured by spotting serial dilutions on YPD plates. After 48 h of incubation at 30 °C, colony-forming units (CFU) were assessed. For the controls, cell suspensions without addition of ethanol were treated in the same way. Survival rate of yeast strains upon exposure to ethanol stress was expressed as the percentage of CFU after ethanol stress and that of control.

### Determination of intracellular free amino acids

For determination of intracellular amino acids, yeast cells were cultured in YPD at 30 °C for 18 h, and then transferred into 50 ml SD medium supplemented with the required amino acids and uracil to a final cell density equivalent to 0.8 of *OD*_600_. Ethanol was added to the cell suspension at different final concentrations and then incubated for 18 h at 30 °C. Yeast cells were harvested from 5 ml of each culture broth by centrifugation at 8000× *g* for 3 min, washed twice with distilled water, and resuspended in distilled water at a final cell density equivalent to 1.0 of *OD*_600_. 0.5 ml of cell suspension was incubated in a boiling water bath for 15 min and then centrifuged at 15,000× *g* for 15 min to remove the cell debris, and the supernatant was collected for amino acids assay by high-performance liquid chromatography (HPLC) with on-line pre-column derivatization. Orthophthalaldehyde (OPA) and 9-formic acid methyl ester of fluorine chlorine (FMOC) were used as the derivatization agents respectively. HPLC analysis was performed on Agilent technology 1260 Infinity and ZORBAX Eclipse-AAA column (3.5 μm, 4.6 × 100 mm) with gradient elution of mobile phase A (40 mM Na_2_HPO_4_, pH 7.8) and mobile phase B (acetonitrile: methanol: water, 45:45:10, v/v) at the flow rate of 2 ml min^−1^. The detailed procedures for on-line pre-column derivatization and HPLC were set according to the Agilent instructions. The OPA derived amino acids were detected at 338 nm, while FMOC derived amino acids were detected at 262 nm with diode-array detector. Amino acid standards (arginine, ornithine, citrulline and proline) were purchased from Sigma-Aldrich (St. Louis, MO, USA). Boric acid buffer, OPA and FMOC were obtained from Agilent (USA). Acetonitrile and methanol (both HPLC grade) were from Fisher Scientific (Leicestershire, UK). Water used for dilutions and for the mobile phase was additionally purified using the Milli-Q water purification system (Millipore, Molsheim, France).

### Analysis of plasma membrane permeability

The cytoplasma membrane permeability was analyzed as described previously^44^, with some modifications. Yeast cells were cultured as described for amino acid assay. In order to investigate the effect of ethanol on membrane permeability, ethanol was added to SD medium at a final concentration of 8% (v/v). After incubation at 30 °C for 0–4 h, yeast cells were harvested by centrifugation, washed twice with 10 mM PBS (pH 6.4) and resuspended in PBS with a final cell density equivalent to 0.1 of *OD*_600_. Propidium iodide (PI) was added to the cell suspension to a final concentration of 15 mg l^−1^. After incubation at 30 °C with PI for 15 min in dark, yeast cells were harvested, washed twice with deionized water and resuspended in the same volume of PBS. The turbidity (*OD*_600_) and the fluorescence intensity (excitation, 485 nm; emission, 635 nm) of the cell suspensions were measured using a F7000 fluorospectrophotometer (Hitachi-hitec, Japan), with PBS as the blank control. The fluorescence intensity was normalized against the cell density (per *OD*_600_) and the level of PI uptake was expressed as the rate of relative fluorescence units (RFU) under ethanol stressed and non-stressed conditions. The PI uptake by yeast cells was also visualized using an Axio Imager A1 fluorescent light microscope (Zeiss, Germany). The dead cell rate was assayed using a BD FACSCalibur flow cytometer (USA) by taking 2 × 10^4^ cells.

### Transmission electron microscopy (TEM) analysis

Changes in morphology of organelles were observed by TEM analysis. After 8 h of incubation in SD medium containing the required amino acids and uracil with or without ethanol, yeast cells were harvested from 5 ml of cultures by centrifugation at 8000 × *g* for 3 min and washed twice with 100 mM PBS (pH 7.2). The cells were resuspended in 5 ml of 2.5% (v/v) glutaraldehyde in PBS and pre-fixed overnight at 4 °C. The cell pellets were washed three times with PBS (20 min for each time) and then resuspended in 0.5 ml of 2% (w/v) KMnO_4_ in PBS. After 2 h of incubation at room temperature, the fixed samples were washed six times with PBS (20 min for each time), resuspended in 0.5 ml of 2% (w/v) uranyl acetate in PBS and incubated for 4 h at room temperature. Upon the completion of uranyl acetate staining, the cell pellets were washed three times with PBS and then performed an acetone dehydration series, in which cells were left in each step for 20 min at concentrations of 30%, 50%, 70%, 80%, 90% and 100%. Yeast cells were collected and embedded in epoxy resin. After polymerization for 8 h at 70 °C, the embedded samples were sectioned with Leica EM UC7 microtome (Germany). The ultrathin sections (thickness, 70 nm) were examined using a JEM-1400 electron microscope (JEOL CO. Ltd., Japan).

### Measurement of reactive oxygen species (ROS) in mitochondria

Intracellular ROS levels in yeast cells were detected by flow cytometry by monitoring the conversion of non-fluorescent dihydrorhodamine 123 (DHR 123, Sigma-Aldrich) to fluorescent rhodamine 123 as previously described^30^. Exponentially growing yeast cells were treated with different concentrations of ethanol at 30 °C for various hours and harvested, washed and resuspended in PBS (pH 7.2) at a final cell density of 1 × 10^6^ cells per milliliter. Then DHR 123 was added to the cell suspensions at the final concentration of 25 μg ml^−1^. After incubation at 30 °C for 2 h with shaking, yeast cells were harvested, washed, and resuspended in 0.5 ml PBS. The fluorescent cells and the median fluorescence intensity were quantified by flow cytometric analysis using a BD FACSCalibur flow cytometer (Becton Dickinson, USA) at low flow rate (12 μl min^−1^) with excitation of 488 nm and the emission fluorescence channel FL1 (533/30 nm) respectively. Typically, 20 000 cells were analyzed per sample. CELLQuest software was used for data acquisition and analysis. ROS levels were represented as the relative units of fluorescence.

## Additional Information

**How to cite this article**: Cheng, Y. *et al*. Protective Effects of Arginine on *Saccharomyces cerevisiae* Against Ethanol Stress. *Sci. Rep.* **6**, 31311; doi: 10.1038/srep31311 (2016).

## Supplementary Material

Supplementary Information

## Figures and Tables

**Table 1:** Intracellular amino acid content of the yeast strains in minimal medium.

Strain	Intracellular amino acid content (mg/g DCW)		
	Arginine	Proline	Ornithine
YS58-V	30.16 ± 0.29b	4.06 ± 0.82	18.21 ± 2.27
YS58-CAR1	17.76 ± 0.10a	5.10 ± 0.36	23.03 ± 1.56
YS58-car1	34.11 ± 0.15c	3.86 ± 0.54	17.72 ± 3.62
YS58-ARG4	35.86 ± 0.21c	4.26 ± 0.43	15.21 ± 2.71

Yeast cells were cultured in SD medium supplemented with the required amino acids and uracil at 30 °C for 18 h. Amino acids were determined as described in Methods. Data are means ± standard deviation (SD) (n = 3). Letters indicate significant differences among different treatments (p < 0.05, Duncan’s multiple-range test).
