# Supplementary material for: Protective Effects of Arginine on Saccharomyces cerevisiae Against Ethanol Stress
Source: Sci Rep. 2016 Aug 10;6:31311. doi: 10.1038/srep31311 (PMC4979094; doi:10.1038/srep31311)
Supplement: Supplementary Information [file srep31311-s1.pdf]

**Protective Effects of Arginine on *Saccharomyces cerevisiae* Against Ethanol Stress**

Yanfei Cheng, Zhaoli Du, Hui Zhu, Xuena Guo, Xiuping He[#]

**Supplementary Table S1. Strains and plasmids used in this study**

| Strains /Plasmids | Genotype or description* | Reference/Source |
|---|---|---|
| **Strains** | | |
| *E. coli* DH5α | *sup*E44 Δ*lac*U169 (φ80*lac*ZΔM15) *hsd*R17 *rec*A1 *end*A1 *gyr*A96 *thi*-1 *rel*A1 | TAKARA |
| *S. cerevisiae* CE25 | *S. cerevisiae* CGMCC 2.1418, wild-type | CGMCC |
| *S. cerevisiae* YS58 | *MATα flo1 leu2-3,112 his4-519 trp1-719 ura3-52* | (32) |
| YS58-V | YS58 (pYCPGA1) | This study |
| YS58-car1 | YS58 (*car1*::*TRP1*) | This study |
| YS58-CAR1 | YS58 (pYGAC1) | This study |
| YS58-ARG4 | YS58 (pYGAA4) | This study |
| **Plasmids** | | |
| YCp50 | *Escherichia coli*-*S. cerevisiae* shuttle vector，$Amp^r$ for *E. coli* and *URA3* for | (36) |

| | | |
|---|---|---|
| | *S. cerevisiae* | |
| pUC18 | *E. coli* vector, Amp$^r$ for *E. coli* | TAKARA |
| pFA6a-kanMX4 | *E. coli*-Yeast shuttle vector, Amp$^r$ for *E. coli* and G418 resistance for *S. cerevisiae* | (35) |
| pYCPG | YCp50 derivative with insertion of G418 resistance cassette between *Sal*I and *Apa*I, Amp$^r$ for *E. coli* and G418 resistance for *S. cerevisiae* | This study |
| pYCPGA1 | pYCPG derivative with insertion of *ADH1* promoter between *Eco*RI and *Bam*HI， Amp$^r$ for *E. coli* and G418 resistance for *S. cerevisiae* | This study |
| pUC18C1 | pUC18 derivative with insertion of *CAR1* between *Kpn*I and *Sal*I, Amp$^r$ for *E. coli* | This study |
| pUc1T1 | pUC18C1 derivative with insertion of *TRP1* between *Afl*II and *Eco*NI to replace 692 bp of partial *CAR1*, Amp$^r$ for *E. coli* | This study |
| pYGAC1 | pYCPGA1 derivative with insertion of coding sequence and terminator of *CAR1* between *Bam*HI and *Sal*I，Amp$^r$ for *E. coli* and G418 resistance for *S. cerevisiae* | This study |

| | | |
|---|---|---|
| pYGAA4 | pYCPGA1 derivative with insertion of coding sequence and terminator of *ARG4* between *Bam*HI and *Sal*I，Amp$^r$ for *E. coli* and G418 resistance for *S. cerevisiae* | This study |

*Amp$^r$, ampicillin resistance

**Supplementary Table S2. Primers used in this study**

| Primer | Sequence (5′-3′)[a] | Purpose |
|---|---|---|
| CAR1-F1 | GCAGGTACCGGTAGCCGCCGAGGGGTCTAAAGAG | PCR of *CAR1* to construct the *CAR1* disruption cassette |
| CAR1-R1 | CGAGTCGACATAGTGTATTGGCAATTAGACGTGG | PCR of *CAR1* to construct the *CAR1* disruption cassette |
| TRP1-F | GTCCTTAAGGTCACCTTACGTACAATCTTGATCC | PCR of *TRP1* |
| TRP1-R | GTGCCTTCTTAAGGCAACAAGTTTGATTCCATTGCGGTG | PCR of *TRP1* |
| KanMX- F | CGCGTCGACAGGCGCGCCAGATCTGTTTAGC | PCR of G418 resistance cassette |
| KanMX-R | GACGGGCCCGCGCCGTTAGTAT | PCR of G418 |

| | | |
|---|---|---|
| | CGAATCGGAC | resistance cassette |
| ADH1-F | GACGAATTCCATAACCGCTAGAGTACTTTGAAGA | PCR of *ADH1* promoter |
| ADH1-R | GACGGATCCTGTATATGAGATAGTTGATTGTATG | PCR of *ADH1* promoter |
| ARG4-F1 | GACGGATCCATGTCAGACGGCACTCAAAAACTAT | PCR of *ARG4* for expression |
| ARG4-R1 | GACGTCGACCGACTTTGGGAGGTTACAATAGTGT | PCR of *ARG4* for expression |
| CAR1-F2 | GACGGATCCATGGAAACAGGACCTCATTACAACT | PCR of *CAR1* for expression |
| CAR1-R2 | GACGTCGACTTTGAGAGCCTCATCGTTACAAAAC | PCR of *CAR1* for expression |
| ACT1-F | CGCTGCTCAATCTTCTTC | qRT-PCR of *ACT1* |
| ACT1-R | GGCTCTGAATCTTTCGTTAC | qRT-PCR of *ACT1* |
| CAR1-F3 | TTGAAATGGCAATGAAAG | qRT-PCR of *CAR1* |
| CAR1-R3 | CCATTATACATTCCTGCTA | qRT-PCR of *CAR1* |
| ARG3-F | CAAGACATCAAGAAGAAGTTAG | qRT-PCR of *ARG3* |
| ARG3-R | ATCAATGGCAGACATAGC | qRT-PCR of *ARG3* |
| ARG4-F2 | CGTCTCTTCCGTATGATTA | qRT-PCR of *ARG4* |
| ARG4-R2 | CCAATTCTGTCTCCGTTA | qRT-PCR of *ARG4* |
| CPA2-F | ATACCACTTACAATGCTACAA | qRT-PCR of *CPA2* |

| | | |
|---|---|---|
| CPA2-R | CAGATGAACCAATACGATAGA | qRT-PCR of *CPA2* |
| CTT1-F | AGTGATATTACATACGCCGC | qRT-PCR of *CTT1* |
| CTT1-R | ACCACTTTCACCACCAACGG | qRT-PCR of *CTT1* |

[a]Restriction sites are underlined.

**Supplementary Figure S1**

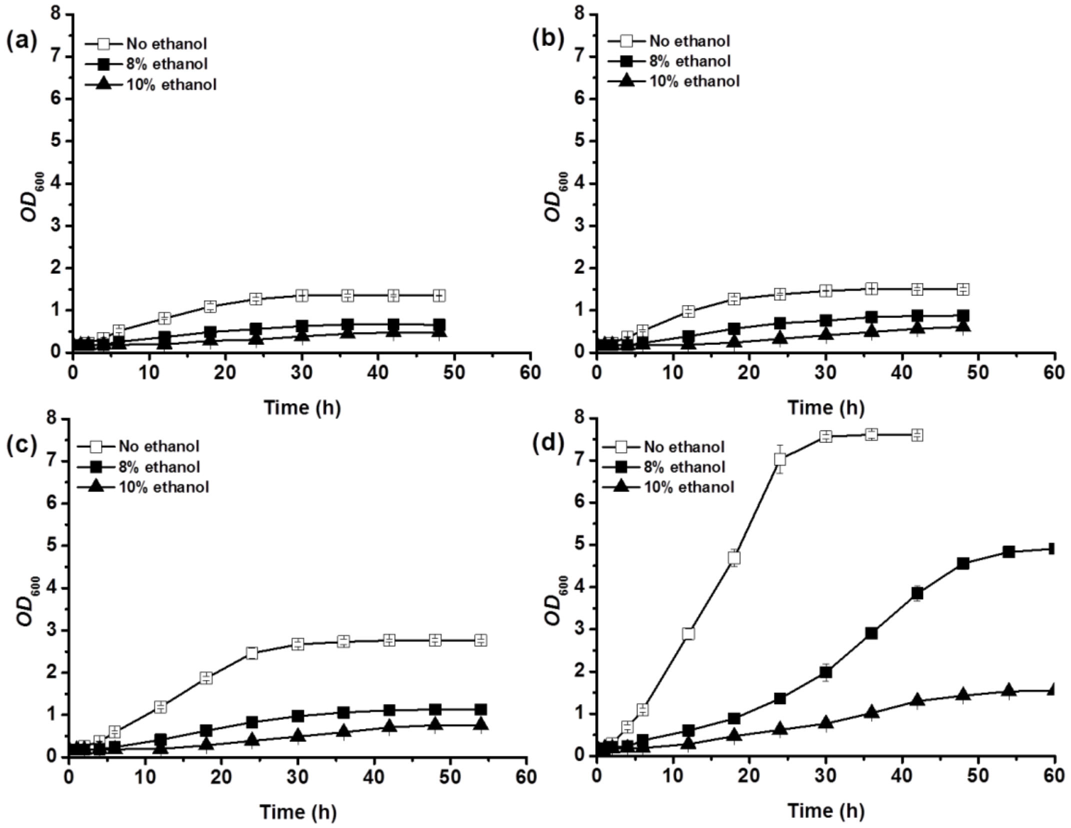


Figure S1 Profiles of cell growth in different media with or without ethanol stress. (a) SD medium supplemented with 30 mg $l^{-1}$ each of uracil, leucine, histidine and 90 mg $l^{-1}$ of tryptophan; (b) SD medium supplemented with 75 mg $l^{-1}$ of uracil, 200 mg $l^{-1}$ of leucine, 60 mg $l^{-1}$ of histidine and 40 mg $l^{-1}$ of tryptophan; (c) SD2 medium containing 2% glucose supplemented with 150 mg $l^{-1}$ of uracil, 400 mg $l^{-1}$ of leucine, 125 mg $l^{-1}$ of histidine and 75 mg $l^{-1}$ of tryptophan; (d) YPD medium. Data are means ± SD (n = 3).
